# Does the contribution of modifiable risk factors on oral health inequities differ by experience of negative life events among Indigenous Australian adults?

**DOI:** 10.1371/journal.pone.0286697

**Published:** 2023-06-08

**Authors:** Lisa Jamieson, Joanne Hedges, Yin Paradies, Xiangqun Ju

**Affiliations:** 1 Australian Research Centre for Population Oral Health, University of Adelaide, Adelaide, South Australia, Australia; 2 School of Human and Social Science, Faculty of Arts and Education, Deakin University, Melbourne, Australia; The University of Queensland, AUSTRALIA

## Abstract

**Objective:**

Although the prevalence of poor self-rated oral health and experience of negative life events among Indigenous adults is high, the contribution of modifiable risk factors is unknown. We aimed to estimate the contribution of modifiable risk factors in poor self-rated oral health among Indigenous Australian adults with high and low experience of negative life events using decomposition analysis.

**Methods:**

The study utilised a cross-sectional design, with data from a large convenience study of Indigenous adults in South Australia. Participants were stratified based on a median split of negative life events in the last 12 months. The outcome was the proportion of fair/poor self-rated oral health (SROH). Independent variables included experience of racism, sex, age, geographic location, car ownership, and time since last dental visit.

**Results:**

Of the 1011 participants, the proportion with fair poor self-rated oral health was 33.5% (95% CI 30.5 to 36.4) and the proportion who had experienced 3+ negative life events in the past 12 months was 47.3% (95% CI 43.7 to 50.9). More than half the contribution in fair/poor self-rated oral health among Indigenous adults with a higher magnitude of negative life events was from experience of racism (55.3%, p<0.001), followed by residential location (19.9%), sex (9.7%) and car ownership (9.8%).

**Conclusions:**

The contributions of modifiable risk factors in poor self-rated oral health among Indigenous adults with different exposures to negative life events differed substantially. Targets to reduce racism will decrease oral health inequities for both groups, however Indigenous adults who have experienced substantial negative life events require additional focus on provision of culturally safe dental care.

## Introduction

Indigenous Australians (those identifying as Aboriginal, Torres Strait Islander or both) experience greater inequities on almost every health and wellbeing indicator compared with non-Indigenous Australians [1]. There is a higher prevalence of non-communicable diseases, including type 2 diabetes, cardiovascular disease, chronic kidney disease and metabolic syndrome [2]. Around three-quarters of Indigenous Australian adults are overweight or obese [3]. The social and emotional wellbeing impacts among Australia’s First Peoples are also high [4]. In 2018–19, around one-quarter of Indigenous Australians reported having a diagnosed mental health or behavioural condition and ‘high or very high’ levels of psychological distress [5]. For many Indigenous Australians, social and emotional wellbeing is a holistic construct encapsulating a sense of belonging, positive interpersonal relationships, strong cultural identity and a belief that life has purpose and value [6]. Poor social and emotional wellbeing is affected by major stressors including removal from family, death of family or close friends, incarceration, unemployment, racial discrimination and everyday stressors that are exacerbated by social disadvantage [4].

These inequities extend to oral health. In Australia’s 2017–18 National Survey of Adult Oral Health, 98 percent of Indigenous adults had one or more missing teeth [7], with earlier estimates suggesting that the prevalence of untreated dental decay among Indigenous adults was 57 percent, compared with 25 percent among non-Indigenous Australians [8]. A frequently cited reason for this inequity is lack of access to culturally-responsive dental providers, together with dental fear, experiences of racism and distrust towards dental health professionals [9].

While the risk factors impacting Indigenous oral health inequities have been identified, there has been no analysis of the difference in risks, and contribution of these differences, between Indigenous adults with high or low experience of negative life events. Nor has there been analysis enabling explicit percentages of risk contribution to be calculated. This is important for policy implications in the allocation of scarce resourcing in the dental public health setting, and for effective oral health promotion initiatives and that account for the broader social and emotional wellbeing environment in which Indigenous Australians live. This study therefore aimed to estimate the contribution of modifiable risk factors in poor self-rated oral health among Indigenous Australian adults with high and low experience of negative life events using decomposition analysis. The hypothesis is that the percentage contribution of each risk factor will differ for Indigenous adults with high experience of negative life events in comparison with their counterparts with low experience of negative life events.

## Methods

Data was obtained from a large convenience sample (n = 1,011) of adults aged 18+ years who identified as Indigenous in South Australia between Feb 2018 and Jan 2020 as part of a broader study investigating HPV infection and oropharyngeal squamous cell carcinoma [10]. The study was governed by an Indigenous Reference Group, with data collected by trained Indigenous research officers. Participants were primarily recruited through Aboriginal Community Controlled Health Organisations (ACCHOs), who were key study stakeholders. After having the study explained and signing informed consent forms, participants were requested to complete a questionnaire (with assistance from study staff if required) that contained information on socio-demographic characteristics, health-related factors, experiences of racism, recent history of negative life events and self-rated oral health.

### Ethical approval

Ethical approval was received from the University of Adelaide Human Research Ethics Committee (H-2016-246) and the Aboriginal Health Council of South Australia’s Human Research Ethics Committee (04-17-729). All participants provided written, informed consent.

### Variables

#### Outcome variable

Self-rated oral health was asked by the question; ‘Would you rate your oral health as’, with response options dichotomised into ‘fair or poor’ and ‘good, very good or excellent’.

#### Exposure variable

Negative life events were captured using a modified form of the Negative Life Events Scale [11]. The specific question asked ‘In the last 12 months, have you or anyone in your family experienced any of the following: ‘incarceration’, ‘domestic violence’, ‘death’, ‘drug/alcohol misuse’, ‘child removal’, ‘psychological distress’ and ‘cultural or spiritual pain’. Response options were ‘yes’ or ‘no’. The median number of negative life events was two, with this variable then dichotomised into ‘≤ 2’ and ‘3+’ negative life events in last 12 months.

### Covariates

**Experience of racism** was assessed using the Measure of Indigenous Racism Experiences (MIRE) [12], which assesses experiences of inter-personal racism across 9 mutually exclusive settings in the last 12 months. The question was: ‘In the last 12 months, have you felt that you have been treated unfairly in any of the following ways because of your racial or ethnic background’? The items evaluate experiences of racism across a range of settings, such as the labour market, housing, health services and education sector. Responses were recorded on a 5-point Likert scale and dichotomized into ‘strongly disagree, disagree, neither agree nor disagree’ and ‘agree and strongly agree’. A participant was considered to have experienced racism if he/she reported being treated unfairly because of their racial or ethnic background in at least one of the 9 settings.

**Demographic characteristics** included age (< 37 years or 37+ years), sex (male or female) and geographic location (metropolitan or non-metropolitan).

**Socioeconomic characteristics** was measured using car ownership from question ‘Do you own a car?’ and two responses (Yes or No).

**Dental behaviour** was measured using time since last dental visit and dichotomised into ‘less than one year ago’ or ‘more than one year ago’.

### Statistical analysis

Descriptive analyses were conducted to examine the distribution of socio-demographic, dental behaviour, experience of racism and negative life events, and the proportion of fair/poor self-rated oral health. Statistically significant differences were denoted by 95% confidence intervals (CI) that did not overlap.

Blind-Oaxaca decomposition analysis was used to evaluate the contribution of demographic (age, sex and geographic location), socioeconomic (car ownership), dental behaviours (last dental visit) and experience of racism (MIRE) factors to changes in oral health (proportion of fair/poor self-rated oral health) between experience of negative life events groups (‘≤ 2’ and ‘3+’). Blind-Oaxaca decomposition analysis is used to calculate the difference in an outcome between two groups by respective differences in the distributions of selected independent variables [13]. All analyses were conducted using the *decompose & oaxaca* command in Stata 17.

## Results

Table 1 shows sample characteristics and cross-tabulations by proportion of fair/poor self-rated oral health. A higher proportion of participants were aged 37 years and above (52%), were female (over 66%), resided in non-metropolitan locations (63%), owned a car (55%), and last visited a dentist greater than 12 months ago (54%). Over half the participants (52%) experienced racism in one or more settings in the last 12 months and almost half (47%) experienced 3 or more negative life events in the last 12 months. The proportion of fair/poor self-rated oral health was 34%. The proportion of fair/poor SROH was higher among older age groups (40%), those who visited a dentist over a year ago (38%) and those who had experienced racism in the last 12 months (39%).

**Table 1:** Sample characteristics and proportion of fair/poor self-rated oral health (SROH) among Indigenous Australian adults (n = 1011).

	% (95% CI)	Proportion fair/poor oral health (95% CI)
**Total**		33.5 (30.5–36.4)
**Age group (Years)**		
**≥ 37**	52.2 (49.1–55.3)	**39.6 (35.4–43.9)**
**< 37**	47.8 (44.7–50.9)	**26.7 (22.7–30.8)**
**Sex**		
Male	33.6 (30.7–36.5)	31.6 (26.7–36.6)
Female	66.4 (63.5–69.3)	34.4 (30.7–38.1)
**Geographic location**		
Non-Metropolitan	62.7 (59.7–65.7)	32.6 (28.9–36.3)
Metropolitan	37.3 (34.3–40.3)	35.1 (30.2–40.0)
**Car ownership**		
No	44.6 (41.5–47.6)	34.0 (29.6–38.5)
Yes	55.4 (52.4–58.5)	33.0 (29.1–37.0)
**Last dental visiting**		
More than one year ago	53.7 (50.6–56.8)	**37.5 (33.4–41.6)**
Less than one year ago	46.3 (43.2–49.4)	**28.9 (24.7–33.1)**
**Measure of Indigenous racism experiences**		
Yes	51.9 (48.8–55.0)	**38.5 (34.2–42.7)**
No	48.1 (45.0–51.2)	**28.1 (24.0–32.2)**
**Negative life events in the last 12 months**		
3+	47.3 (43.7–50.9)	36.2 (31.1–41.3)
≤ 2	52.7 (49.1–56.3)	33.9 (29.1–38.6)

The distribution of negative life events in the last 12 months is shown in Table 2. Over 85 percent of participants had experienced one or more negative life events. Almost two-thirds (65%) had experienced death of a close friend or family member. Over half (56%) had experienced psychological distress and 43% had experienced drug and/or alcohol misuse. Around one-fifth (19.7%) of participants had experienced one negative life event in the last 12 months, 15% had experienced two and 14% had experienced three. Almost two-thirds (64%) of participants had experienced 3 or more negative life events, and almost one-quarter (23.3%) had experienced 5 or more negative life events in the last 12 months.

**Table 2:** Distribution of responses to the negative life events scale* among Indigenous Australian adults (n = 1011).

	%	Cumulative %
1: Incarceration	27.5	
2: Domestic violence	30.4	
3: Death	65.0	
4: Drug/alcohol abuse	42.5	
5: Child removal	17.3	
6: Psychological distress (depression/anxiety)	55.7	
7: Cultural/spiritual pain	33.6	
8: Other	22.9	
No negative life event	15.3	15.3
1 negative life event	19.7	35.2
2 negative life events	15.0	50.2
3 negative life events	14.1	64.3
4 negative life events	12.5	76.7
5 negative life events	7.9	84.6
6 negative life events	8.6	93.2
7 negative life events	4.8	98.0
8 negative life events	2.0	100.0

* The question asked was: ‘In the last 12 months please tick if you or anyone else in your family has experienced any following: (response options above)’

The distribution of experience of racism in the last 12 months is shown in Table 3. Nearly one-third (31.6%) had experienced racism in law enforcement, 22% had experienced racism in government service provision and almost one-fifth (18.8%) had experienced racism in employment. Around 16% had one experience of racism in the last 12 months, 9% had two experiences and 7% had three experiences. Just over one-fifth (20.1%) had four or more experiences of racism in the last 12 months.

**Table 3:** Distribution of responses to the Measure of Indigenous Racism Experiences scale* among Indigenous Australian adults (n = 1011).

Where racism was experienced:	%	Cumulative %
1: Employment	18.8	18.8
2: Domestic	14.1	32.9
3: Educational/academic	17.1	50.0
4: Recreational/leisure	15.7	65.7
5: Law (enforcement)	31.6	97.3
6: Health care	17.6	114.9
7: Government service provision	22.0	136.9
8: Public settings	17.2	154.1
9: Other	24.6	178.7
No experience of racism	48.1	48.1
1 experience of racism	16.3	64.4
2 experiences of racism	8.5	72.9
3 experiences of racism	7.0	79.9
4 experiences of racism	5.2	85.1
5 experiences of racism	4.8	89.9
6 experiences of racism	3.4	93.3
7 experiences of racism	2.2	95.5
8 experiences of racism	1.5	97.0
9 experiences of racism	3.0	100.0

* The question asked was: ‘In the last 12 months, have you felt that you have been treated unfairly in any of the following ways because of your identity as an Aboriginal and/or Torres Strait Islander person’? (response options above)’

The decomposition of negative life events changes (from 2 or less to 3 or more in the last 12 months) in proportion of fair/poor self-rated oral health shows that approximately 85% of the increase was explained by the change in sociodemographic characteristics, and experience of racism (Table 4). More than half the contribution was from experience of racism (55%), followed by residential location (nearly 20%), car ownership and sex (approximately 10 percent each).

**Table 4:** Decomposition of the change in the proportion of fair/poor self-rated oral health (SROH) among Indigenous Australian adults.

Proportion of SROH (negative life events 3+)	36.2%			
Proportion of SROH (negative life events ≤ 2)	33.9%			
Due to endowments (E)	0.025			
Due to coefficients (C)	0.016			
Due to interaction (CE)	-0.011			
Explained %	84.4%			
Unexplained %	15.6%			
Explanatory variables	E	C	E (Neumark^a^)	Proportion explained (%)
Age group	0.001	-0.178	0.001	5.44
Sex	0.000	-0.026	0.001	9.69
Geographic location	0.003	-0.003	0.003	19.88
Car ownership	0.001	0.012	0.001	9.75
Last dental visit	-0.005	0.050	-0.006	0
Experience of racism	0.012	0.140	0.020	**55.25
Total	0.019	0.016	0.020	100

Notes

**p-value<0.01

*p-value<0.05.

^a^ Coefficients obtained from the pooled data regression [14]. The coefficients were obtained from the pooled data regression. E (and E Neumark), C and CE show the contribution attributable to the gaps in endowments (E), the coefficients (C) and due to the interaction (CE). In this study, the gap in endowments accounts for the greatest bulk of the gap in outcomes. Proportion explained: related to change in endowments, attributable to experience of negative life events changes in the magnitude of the explanatory variables.

Unexplained: related to change in coefficients.

## Discussion

This study tested the hypothesis that the contribution of socio-demographic, economic and behavioural risk factors on fair/poor self-rated oral health would differ for Indigenous adults with high experience of negative life events in comparison with their counterparts with low experience of negative life events. The hypothesis proved to be true, with racism being the risk factor with the most overwhelming contribution. The findings have important policy translation implications, as they indicate that while targets to reduce structural and personal racism, in its various guises, will reduce oral health inequities for all Indigenous Australians, an additional focus is particularly required for Australia’s First Peoples with recent experience of negative life events (with direct implications on social and emotional wellbeing).

It is important to highlight the overwhelmingly high proportion of both racism (52%) and negative life events (3+, 47%) experienced by Indigenous participants in our study. Markwick and colleagues, in a population health survey in Victoria, Australia, estimated that 17% of their Indigenous participants experienced at least one episode of racism the previous year compared with 5% of non-Indigenous participants [15]. In research involving 1,033 Indigenous Australians, 38% reported being treated unfairly because of their Indigenous background, 44% reported hearing racial slurs in the workplace, and 59% reported receiving comments about the way they look or ‘should’ look as an Indigenous person. Just over one-quarter (28%) reported working in "culturally unsafe workplaces" [16]. Our estimates are far higher than those reported in these recent surveys. To the best of our knowledge, there are no estimates of negative life events at a population level for Indigenous Australians. However, in the 2014–15 National Aboriginal and Torres Strait Islander Social Survey, 22% of participants had experienced physical violence in the last 12 months, with 8 percent experiencing physical violence on more than one occasion [5]. Fifteen percent had been arrested in the last five years and 9 percent had been incarcerated in their lifetime.

Just over one-third of participants self-rated their oral health as fair or poor, compared with 24 percent reported in the 2017–18 National Survey of Adult Oral Health [17]. The self-rated oral health estimates were more favourable than other populations involving Indigenous Australians. For example, in a convenience sample of Indigenous adults from the Northern Territory, the proportion of fair/poor self-rated oral health was 52%, and among a cohort of women pregnant with an Aboriginal child in South Australia, the proportion of fair/poor self-rated oral health was 54 percent [18].

In recent years, there has been concerted efforts from dental professional and governance bodies to name and address racism in the dental sector in Australia. This includes in dental public health settings [19], research projects [20], funding agencies [21] and regulatory authorities including the Australian Health Practitioner Health Regulation Agency [22]. Internationally, there has been substantial effort to increase the profile of anti-racist frameworks in dental school curricula [23], with this movement also obtaining momentum in Australian dental schools.

Our findings emphasize the inherent uniqueness of the social composition and life experiences of many Indigenous Australians that makes Indigenous Australians so much more vulnerable to downstream consequences including poor oral health than their non-Indigenous peers. This is distinct from differences in social advantage alone. It is telling that experience of racism was the determinant having the most impact on the outcome (55%), with the effect of geographic location also being substantial (20%). However, whilst policies to reduce racism are increasing in their scope and remit across a multitude of services, and the challenges with regional and remote dwelling are increasingly recognised by government agencies, the sheer magnitude of negative life events experienced by many Indigenous Australians is a difficult challenge to address. This is, in large part, because of the layering of oppressions experienced by many Indigenous Australians, and the intersections, complexities and early age of onset of these commencing, that continue (and multiply) across the life course.

Study limitations include the design being cross-sectional, meaning no causal inferences can be implied. In addition, because a convenience sample was used some bias sources to compose the study population were not controlled. Specifically, the magnitude of outcomes and exposures, and the risks of over/underestimation including the consequences to associations observed, were not accounted for in the analysis. In conclusion, our study provides new evidence on the magnitude of oral health inequities experienced by Indigenous Australians with differing exposure to negative life events, and the contributions of modifiable risk factors including experiences of racism. A deeper understanding of the insidious and pervasive nature of negative life events for Indigenous Australians, and efforts by policy makers and all health professionals are required to reduce preventable oral health inequities. This would go some way to addressing key priority areas as outlined in the 2015–2024 National Oral Health Plan [24].
